# Histopathology of Pseudoxanthoma Elasticum and Related Disorders: Histological Hallmarks and Diagnostic Clues

**DOI:** 10.6064/2012/598262

**Published:** 2012-07-25

**Authors:** Mohammad J. Hosen, Anouck Lamoen, Anne De Paepe, Olivier M. Vanakker

**Affiliations:** ^1^Center for Medical Genetics, Ghent University Hospital, De Pintelaan 185, 9000 Ghent, Belgium; ^2^Department of Genetic Engineering and Biotechnology, Shahjalal University of Science and Technology, Sylhet 3114, Bangladesh

## Abstract

Among ectopic mineralization disorders, pseudoxanthoma elasticum (PXE)—a rare genodermatosis associated with ocular and cardiovascular manifestations—is considered a paradigm disease. The symptoms of PXE are the result of mineralization and fragmentation of elastic fibers, the exact pathophysiology of which is incompletely understood. Though molecular analysis of the causal gene, *ABCC6*, has a high mutation uptake, a skin biopsy has until now been considered the golden standard to confirm the clinical diagnosis. Although the histological hallmarks of PXE are rather specific, several other diseases—particularly those affecting the skin—can present with clinical and/or histological characteristics identical to or highly resemblant of PXE. In this paper, we will summarize the histopathological features of PXE together with those of disorders that are most frequently considered in the differential diagnosis of PXE.

## 1. Introduction

Ectopic calcification is a pathologic deposition of calcium salts or inappropriate biomineralization in soft tissues [1], resulting in the formation of osseous material in soft tissues including the lungs, eyes, arteries, and skin. Ectopic mineral deposits in the extracellular matrix (ECM) of the cell can result in fragmentation of connective tissue components, such as the elastic fibers.

**Figure 1:**
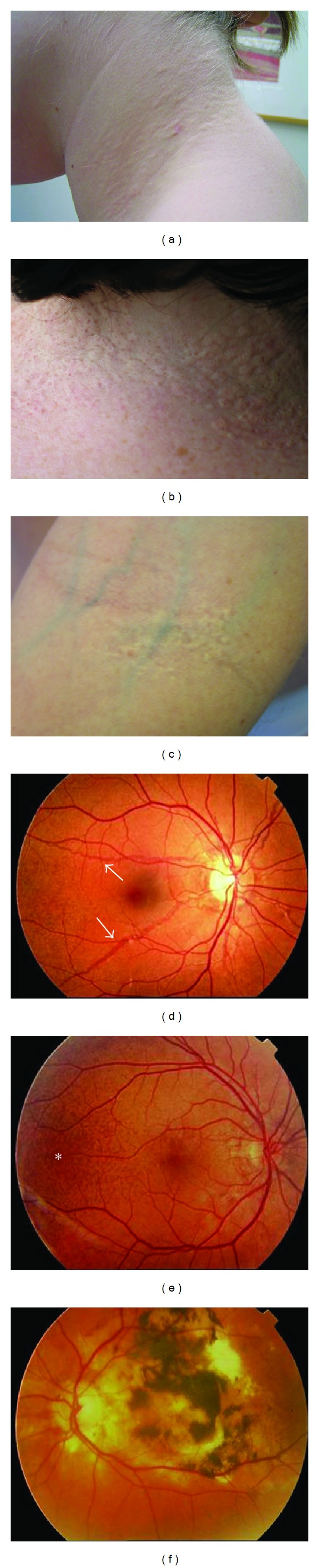
Cutaneous and ophthalmological features of PXE. Skin lesions include yellowish papular lesions in neck and flexural areas (a–c), often coalescing into larger plaques (b). In fundo, angioid streaks (d, arrowed) can be observed, as well as a mottled aspect of the fundus called peau d'orange (e, asterisk). Following neovascularisation, subretinal haemorrhage and vision loss can occur (f).

The paradigm of ectopic mineralization disorders is pseudoxanthoma elasticum (PXE, OMIM# 264800). This rare hereditary connective tissue disorder affects the ECM in different organs and—because of its molecular and etiopathological characteristics—has a unique position among the connective tissue diseases [2]. It is characterized by dystrophic mineralization and fragmentation of elastic fibers and causes dermal (papular lesions in flexural areas), ocular (angioid streaks, subretinal neovascularization, and haemorrhage), and vascular symptoms (coronary and peripheral vascular disease) (Figure 1). PXE is caused by mutations in the *ABCC6 *(ATP-binding cassette subfamily C member 6; OMIM# 603234) gene, encoding a transmembrane transporter protein, the substrate of which is currently unknown. Moreover, the exact relation between the ABCC6 transporter and the elastic fiber abnormalities remains unclear.

Though the pathophysiology of PXE is still ill-defined, two main concepts have been proposed to explain the consequences of *ABCC6* mutations, respectively, coined as the metabolic and cellular hypothesis. The metabolic hypothesis results from the observation that ABCC6 is expressed primarily in the liver and kidney, and to much lesser extent in the affected organs, and suggests that a decrease of ABCC6 transport activity in the liver results in altered plasma levels of one or more substrates [2]. The cellular hypothesis of PXE is based on the observation that PXE fibroblasts are suffering from a mild chronic oxidative stress due to imbalance between the production and degradation of oxidative stress species as a consequence of ABCC6 deficiency [3]. 

Several other dermatological or system diseases with skin manifestations can have similar clinical and/or histological features as PXE. These include among others the PXE-like syndrome with coagulation deficiency, elastosis perforans serpiginosa, and haemoglobinopathies such as thalassemia and sickle cell disease. In this paper, the histological hallmarks of PXE and PXE-related disorders will be discussed, both in humans and murine models, focussing on those characteristics which can differentiate them from one another. For each disease, the histopathological clues will be shortly summarized and—if available—immunohistochemical findings relevant to the pathogenesis of disease will be considered.

## 2. Pseudoxanthoma Elasticum

### 2.1. Histopathology in Humans

The primary histological feature of PXE is degeneration of elastic fibers that undergo progressive mineralization and fragmentation resulting in a histological image pattern known as elastorrhexis [4, 5]. These alterations can be observed by light microscopy (LM) and electron microscopy (EM) in the main affected organs of PXE patients including skin, retina and blood vessels but also in other tissues which contain elastin. The latter include the urinary system (kidney, bladder), the gastrointestinal tract (oesophagus, intestines), and the pulmonary system (trachea, lung). The alterations in these systems, although often widespread and associated with collagen abnormalities, are, however, usually very small which could account for their lack of clinical significance. 

Although variation can be noted in the histological findings in the different affected tissues, the chain of degenerative events is always alike. One of the intruiging questions was whether the primum movens was the calcification or fragmentation. Calcified fibers are prone to degradation, while fragmented fibers become more easily calcified. EM studies have reported calcification in elastic fibers that appear normal as the first pathological sign in young individuals, allowing to put forward a pathological cascade as follows [6–8]. Initially, mineralization of the elastic fiber is seen as a central core of electron density on EM, with core density increasing as mineralization continues. Prior to fragmentation, the elastic fibers will develop holes, where the central portion of the core disappears or spontaneously fades. Finally, the fibers become maximally calcified, followed by fragmentation. 

Two main kinds of calcification have been described: one composed of hydroxyapatite and the other of CaHPO_4_ [9]. Other mineral precipitates, such as iron, phosphate, and carbonate, have also been identified in altered elastic PXE fibers [10–13]. 

A series of studies have described abnormalities of other ECM components, such as collagens and proteoglycans. Collagen flowers, a sign of abnormal collagen fibrillogenesis, are—although commonly found in PXE—also highly aspecific. Abnormal amounts of proteoglycans are localized nearby and within mineralized elastic fibers and abnormal amounts of GAGs, as well as alterations in their synthesis and deposition have been detected in PXE patients [14–17]. Moreover, PXE cells have been shown to produce proteoglycan species with altered properties, such as stronger polyanion properties, increased hydrodynamic size, abnormal hydrophobic actions, and different content and distribution of heparan sulphate, indicating an abnormal proteoglycan metabolism [17, 18]. In urine of PXE patients and carriers, both decreased and increased concentration of GAGs has been observed [18, 19]. Although no straightforward explanation exists for these discrepant findings, Maccari et al. found three distinguishing differences of urinary GAGs in PXE: the chondroitin sulphate/heparin sulphate ratio (which is lower), the 4-sulphated/6-sulphated chondroitin sulphate ratio (which is lower), and the high degree of chondroitin sulphate sulfation [4]. 

 Baccarani-Contri et al. showed that elastic fibers have enhanced expression of normal constitutive proteins (e.g., vitronectine), but also accumulated aberrant matrix proteins known for their high affinity for calcium and involvement in mineralization processes (e.g., alkaline phosphatase, bone sialoprotein, osteonectin) [16]. 

#### 2.1.1. Histopathology of the Skin

**Figure 2:**
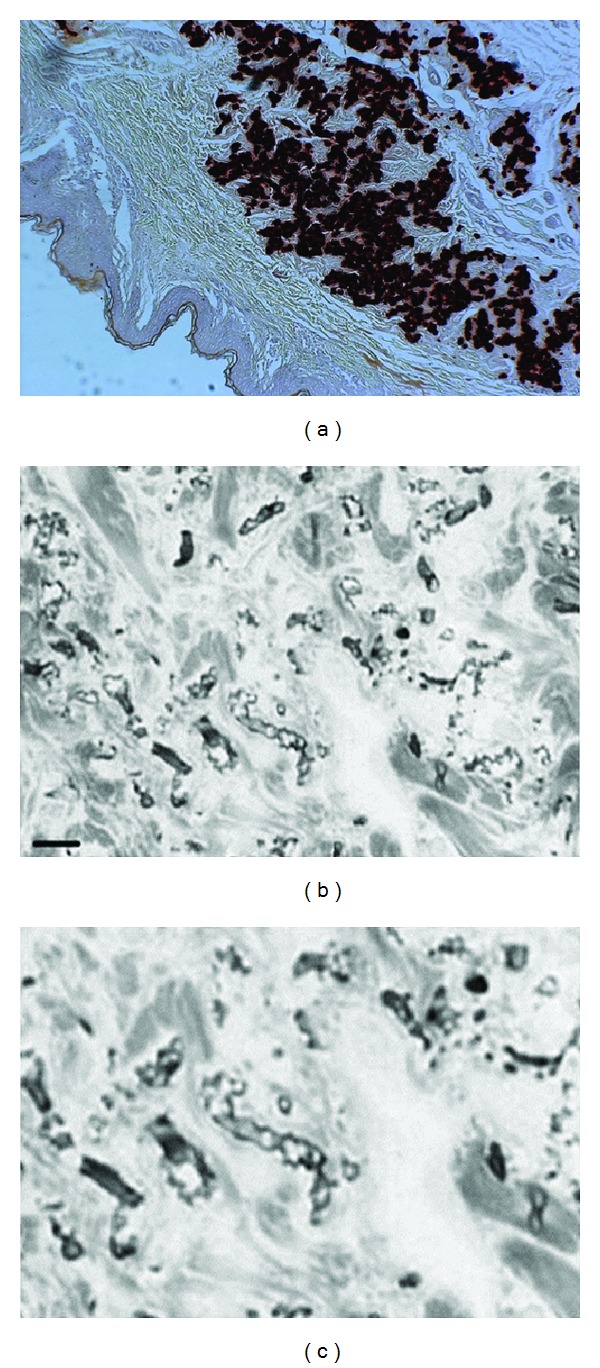
Histological characteristics of PXE skin lesions with fragmentation and calcification of middermal elastic fibers on Alizarin Red Staining (a). Electronmicroscopy reveals fragmentation of elastic fibers (b) with the calcification being present in the core of the elastic fiber (c).

Elastic fibers in the mid-dermis are typically polymorphous, mineralized and fragmented, while those in the papillary dermis and deep dermal layers have a normal morphology. Light microscopy using von Kossa or Alizarin Red calcium stains reveal middermal clumps of calcified and fragmented elastic fibers (Figure 2(a)). By EM, two types of mineralization can be noted: fine deposits in the center of the fiber and bulky precipitates deforming and breaking the fibers (Figures 2(b) and 2(c)) [20–22]. In these dermal mineralized areas, deposits of thread-like material, collagen flowers, and collagen fibrils of irregular diameter are present in most patients. Fibroblasts are often numerous, with hypertrophy and dilatation of the endoplasmatic reticulum [20]. Near the mineralized areas, macrophages are abundant. Interestingly, ultrastructural elastic tissue alterations can be observed in both lesional and clinically noninvolved skin, while the other ECM changes are only seen in clinically involved skin in vicinity of aberrant elastic fibers [23]. In rare cases, the dermal calcification can lead to ossification [24]. Interestingly, this has always been noted in those patients with significant cutis laxa. Of note, the dermal EM alterations are certainly not specific for PXE; they can also be seen in other inherited diseases of the ECM and normal skin aging [23]. Therefore, LM evaluation remains essential for the diagnosis of PXE. In this regard, the quality of the skin biopsy—particularly that it is a full-thickness specimen—as well as its location is essential for a reliable result. Indeed, contrary to EM findings, LM aberrations in nonlesional skin can be very mild or absent. 

#### 2.1.2. Histopathology of the Eye

**Figure 3:**
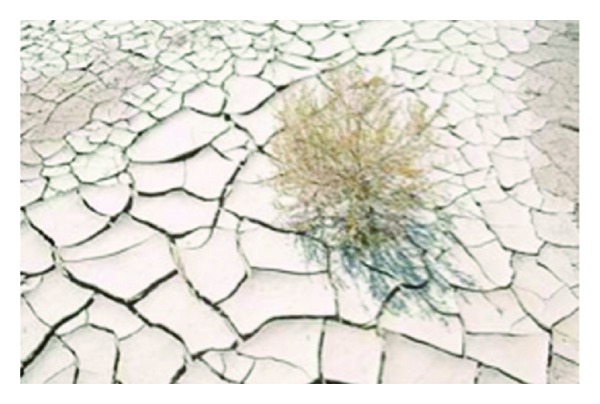
Graphic representation of BrM degradation (crack formation) and subsequent growth of bloodvessels through the breaks.

PXE typically affects elastic fibers in Bruch's Membrane (BrM), a thin connective tissue layer separating the retina from the choriocapillaris. The changes in BrM are apparently identical to those seen in the middermis, with similar calcium deposits onto and clumping of fragmented elastic fibers. As a result, the barrier membrane will no longer have a smooth surface, but will start to exhibit breaks, making it possible for choroid vessels to grow towards the inner retina (Figure 3). The start of this degradation is characterized by discontinuities in the middle elastic layer and loss of RPE pigment granules. In a next stage, full thickness breaks in combination with atrophy of the overlying RPE and photoreceptor cells are observed plus ruptures of the underlying choroid. In some cases, herniation of choroidal fibrillar collagen tissue and choriocapillaris into the breaks in BrM can be seen, separating this membrane. 

#### 2.1.3. Histopathology of the Cardiovascular System

In PXE patients, both the myocardium and pericardium show elastic fiber mineralization and collagen flowers, resembling the mid-dermal alterations [4]. Similar findings can be observed in nearly all small and medium-sized bloodvessels, including intestinal vessels, although it has been observed that alterations are not homogeneously distributed in the vessel wall. Alterations are most prominent close to the adventitia [4]. 

In contrast to the dermis and retina, vascular elastic fibers tend to form aggregates of thin strands of amorphous elastin, which replaces the internal elastic lamina, for example, in the aorta. These degenerative changes may be accompanied by various degrees of intima thickening due to a patchy proliferation pattern of the fibroelastic components [25]. These vascular changes show great comparison with Mönckeberg-type arteriosclerosis and the idiopathic type of generalized arterial calcification in infancy (IACA). The former is a type of focal calcific arteriosclerosis in the elderly, while IACA is a dramatic variant of arterial myoelastic degenerative tissue calcifications, caused by mutations in the *ENPP1* gene (OMIM# 173335) and leading to intrauterine death or lethality within the first months of life. While recent advances in deep phenotyping of the vascular implications of PXE have led to the suggestion of a unique vasculopathy in PXE, it remains interesting that recently *ENPP1* mutations have been found in classic PXE patients, while some IACI patients were discovered to harbor *ABCC6* mutations. This underlines that these disorders are in fact part of the same spectrum of diseases [26, 27]. 

Also the venous system, more specifically the vena cava, can be affected at the ultrastructural level in PXE [4]. 

### 2.2. Histopathology of the Murine Model

**Figure 4:**
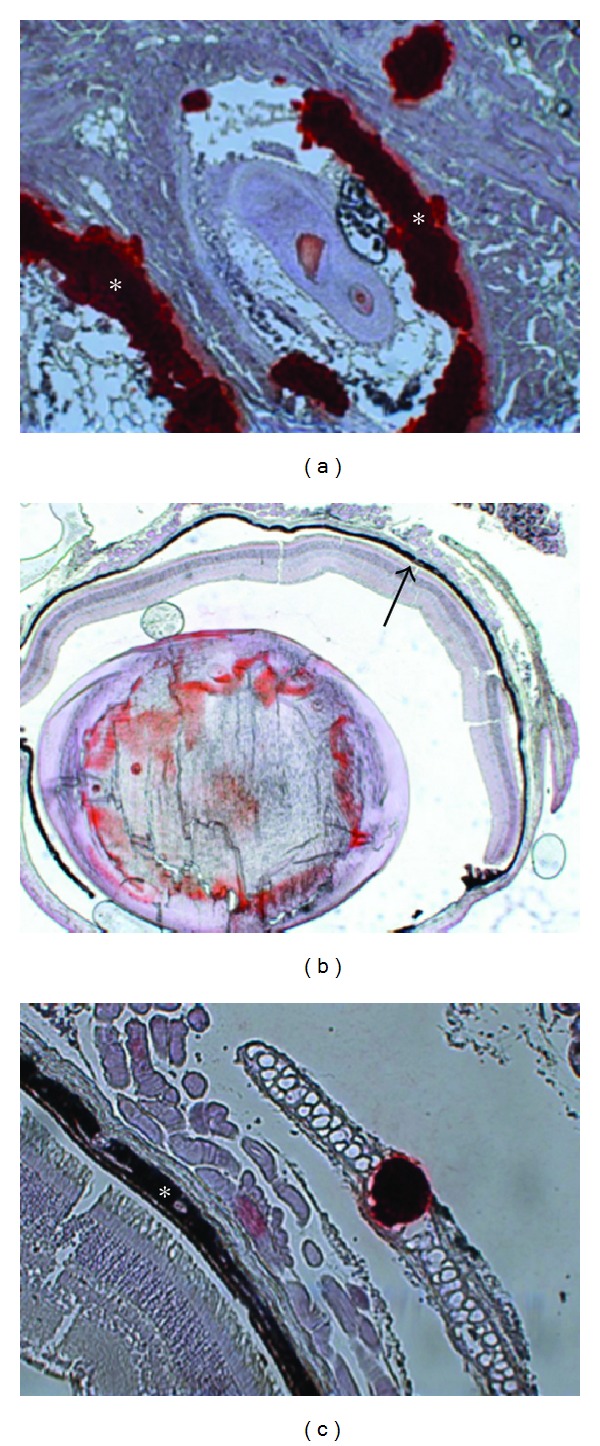
Histopathology of the murine PXE model using Alizarin Red and von Kossa staining with extensive mineralization in the connective tissue sheet of the whiskers (a, asterisk, ×20 and mineralization of Bruch's Membrane of the eye (b arrowed, c asterisk, ×10 and ×20).

The murine PXE model is a transgenic mouse model generated by targeted ablation of the mouse *Abcc6* gene [28]. Light and electron microscopy observations of Accc6-/-mice revealed that KO mice spontaneously developed calcification of elastic fibers in blood vessel walls and in Bruch's Membrane in the eye, while no clear abnormalities were seen in the dermal ECM, possibly due to the different distribution of elastic fibers in murine dermis compared to humans (Figure 4) [28]. Mineralization can affect both elastic structures and collagen fibers [29]. Calcification of blood vessels was most prominent in small arteries in the cortex of the kidney [28]. Klement et al. also found profound mineralization of several tissues, including skin in the *Abcc6 *null mice [29]. Interestingly, calcification in the murine model started in the connective tissue sheet around the whiskers, and this feature has been used as a biomarker for calcification in murine PXE since (Figure 4). 

### 2.3. Immunohistochemical Pathogenetic Clues in PXE

Soft tissue mineralization is a complex process, the exact mechanisms of which have not yet been completely elucidated. However, a significant overlap has been observed in several studies with osteogenesis and several proteins involved in bone metabolism have been implicated in soft tissue calcification. Besides the many propagators of calcium precipitation, ectopic mineralization tends to be more frequently due to a disruption of calcification inhibitors. The latter include the endogenous inhibitor matrix Gla protein (MGP), the inducible inhibitor osteopontin (OPN), and the systemic circulating inhibitor fetuin-A [30–32]. 

**Figure 5:**
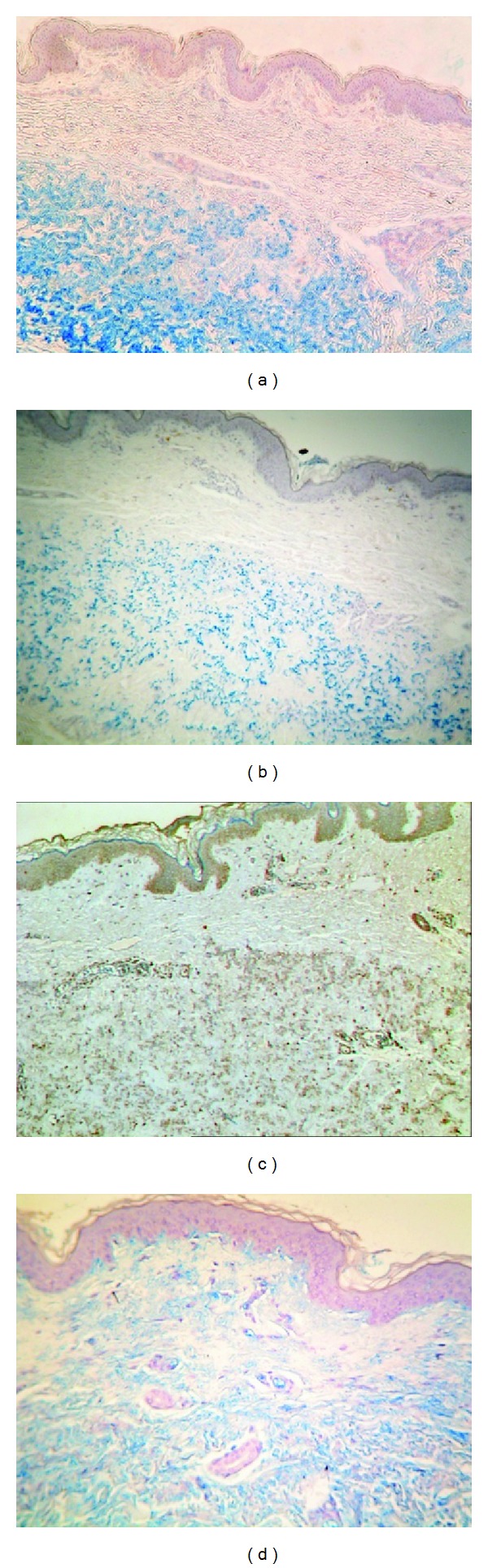
Immunohistochemical findings in PXE, showing excessive middermal staining of carboxylated (a, ×20) and uncarboxylated (b, ×20) MGP, as well as osteocalcin (c, ×20) and fetuin-A (d, ×20).

In PXE patients, the role of soft tissue calcification inhibitors has been studied by establishing serum concentration of osteocalcin (OC), fetuin-A and MGP. It was observed that the total amount of OC was decreased in patients [33]. PXE patients also have a significantly lower serum level of both MGP and fetuin-A than controls [34–36]. Dermal fibroblasts of PXE patients produce less of the *γ*-carboxylated form of MGP compared to controls, suggesting that these cells also play a role in ectopic calcification in PXE [34]. Immunohistochemistry on PXE dermis revealed significant middermal staining of carboxylated and uncarboxylated MGP, as well as osteocalcin and fetuin-A (Figure 5). 

In the murine knockout model for PXE, a reduction in fetuin-A serum levels was found compared to control, albeit that there was considerable variability between the individual mice [37, 38]. On IHC of the whiskers in abcc6-/-mice, MGP colocalized with mineralization but in its inactive or under-carboxylated form [38]. 

## 3. PXE-Like Disease with Coagulation Deficiency

**Figure 6:**
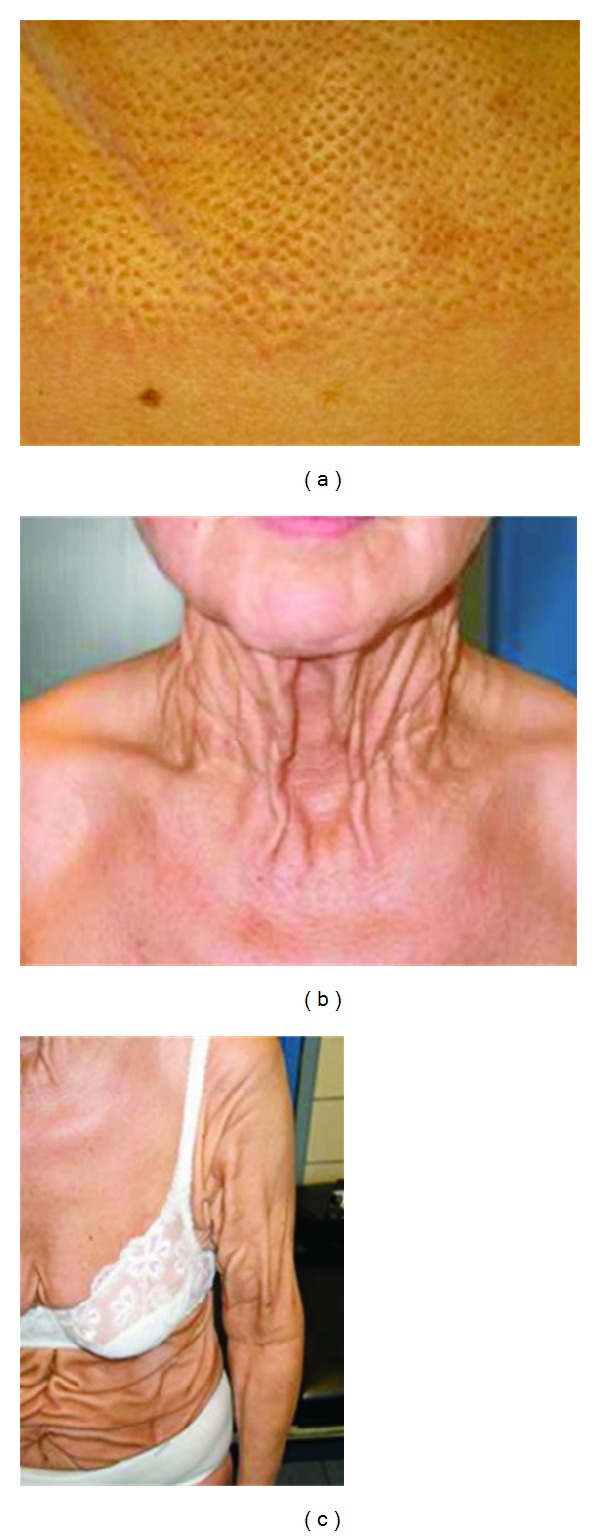
Clinical features of the PXE-like disease with coagulation factor deficiency. Typical cutaneous peau d'orange lesions (a) may be the first sign. Natural history reveals an evolution towards thick redundant skin folds in the neck (b), the flexural regions and beyond (c).

In PXE-like disease with coagulation deficiency (OMIM# 610842), patients initially present with clinical features, which cannot be distinguished from classic PXE. However, the natural history of this disease is quite different with progression of the skin lesions towards thick and leathery skin folds beyond the flexural areas (Figure 6) [39]. Contrary to PXE, ophthalmological and cardiovascular manifestations usually remain mild. In addition, these patients have a deficiency of the vitamin-K- (VK-) dependent coagulation factors (factor II, VII, IX, and X), though most often this remains asymptomatic and is a coincidental finding. The disease is caused by loss-of-function mutations in the *GGCX* gene (OMIM# 137167), encoding a gamma-carboxylase which in the liver is responsible for the activation of VK-dependent coagulation factors. In peripheral tissues, it activates several inhibitors of mineralization, including MGP. 

The histopathology of this PXE-like disease is indistinguishable from classic PXE on LM. However, EM reveals several specific characteristics, which can be used in the differential diagnosis, particularly in young individuals in whom the clinical presentation may still be identical to classic PXE. 

### 3.1. EM Histopathology

**Figure 7:**
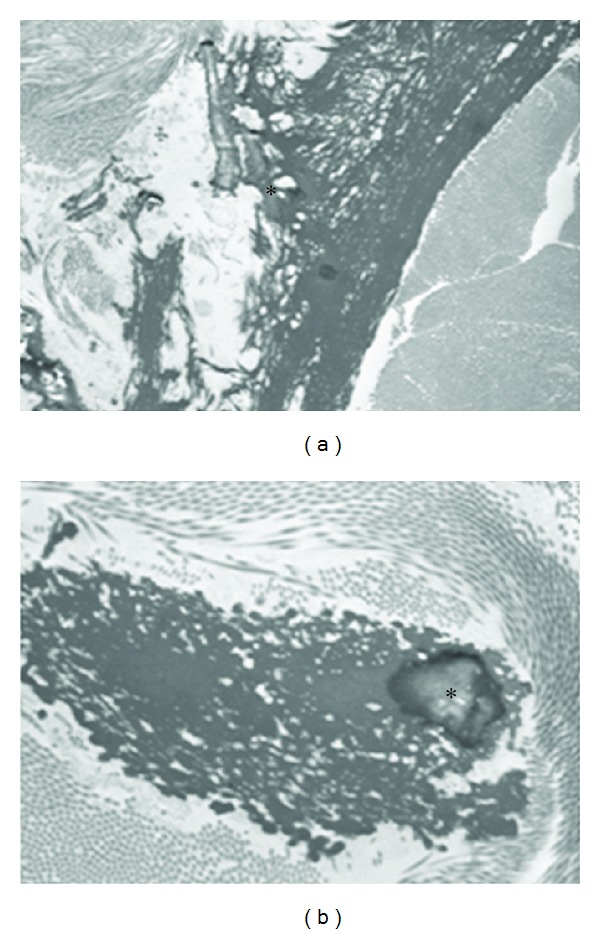
EM findings in the PXE-like disease with coagulation factor deficiency. The elastic fiber network displays a ragged aspect (a, asterisk), while mineralization can be observed at the periphery of affected elastic fibers (b, asterisk).

The overall appearance of the elastic fiber network has a rather “ragged” aspect, as if someone pulled at it [39]. Although mineralization is most prominent in the middermis and does not affect all elastic fibers, the morphology on EM is different from classic PXE. Indeed, they are often made of aggregates of distinct strands of elastin and confined to the periphery of the elastic fiber, whereas PXE mostly affects the elastic fiber core. Also, electron-dense crystal-like bodies can be seen in the central core of mineralized fibers in PXE-like patients, whereas in PXE this feature is not encountered (Figure 7). 

### 3.2. Immunohistochemical Findings

The immunohistochemical staining for carboxylated and uncarboxylated MGP, as well as for osteocalcin and fetuin-A, were similar to what was observed in PXE. On EM, uncarboxylated MGP was more abundant and located in the core of elastic fibers, whereas carboxylated MGP resided at the border of the mineralized areas. 

## 4. PXE Phenocopies Associated with Haemoglobinopathies

**Figure 8:**
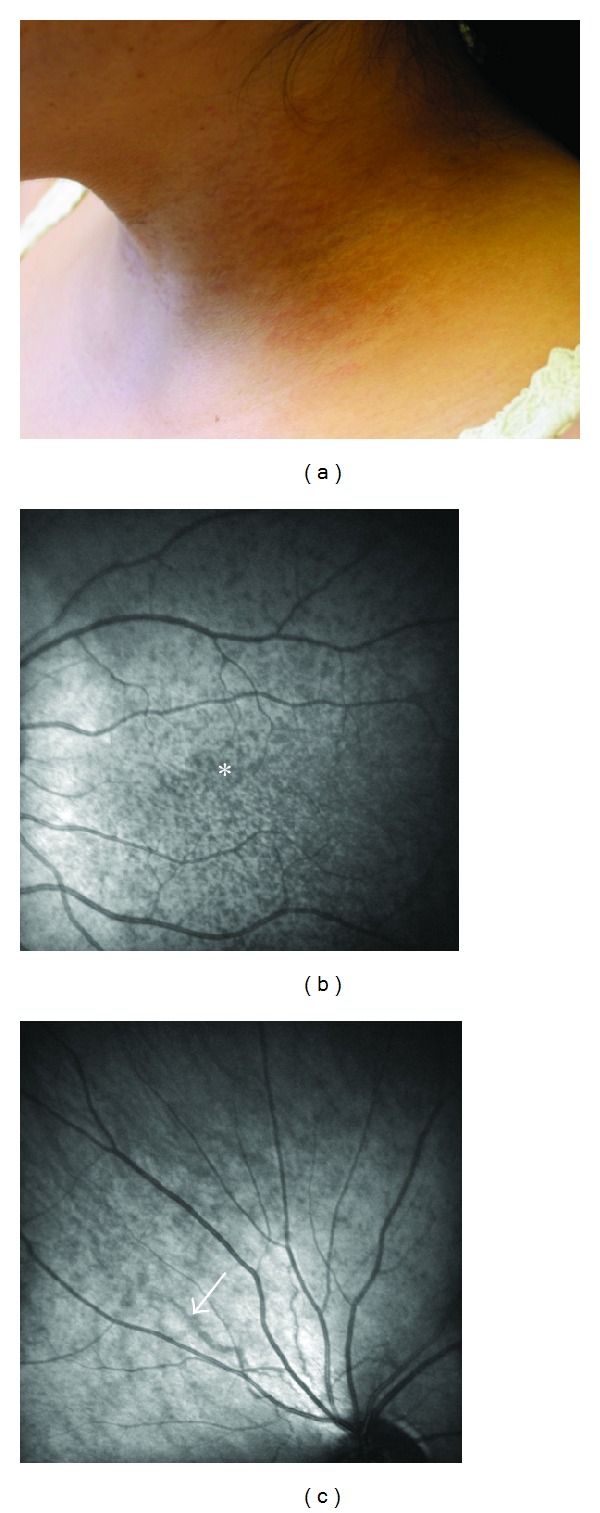
Clinical features of a patient with beta-thalassaemia and PXE lesions. The small papules on the lateral side of the neck are indistinguishable from hereditary PXE (a). In fundo, peau d'orange (b, asterisk) and angioid streaks (c, arrowed) can be seen as in classic PXE (HRA infrared imaging).

Patients with inherited haemoglobinopathies, most often thalassaemias but also sickle cell disease, may have elastic tissue changes and clinical features closely resembling—if not identical to—PXE. The skin, eye, and cardiovascular symptoms are indeed indistinguishable from PXE—except for their age of onset which is usually later in life—despite that none of these patients carry *ABCC6 *mutations (Figure 8) [40–49]. These clinical findings are not anecdotal in haemoglobinopathy patients: out of a cohort of 100 patients with major or intermediate beta-thalassaemia, 26 patients had angioid streaks and/or skin lesions [46]. While one hypothesis might be that this is related to the pathophysiology of the primary haemoglobinopathy (and as such “acquired”), the identical histological findings in these patients suggest that either a pathway is involved which is independent of ABCC6 or that the largely unknown ABCC6 pathway is affected more downstream. As a result, a haemoglobin electrophoresis should be considered in the standard workup of every patient presenting with clinical and/or histological features resembling PXE. 

## 5. Dermatological Diseases with (Near-) Identical Skin Lesions

Disorders with a phenotype showing significant resemblance to PXE and resulting from elastic fiber abnormalities are usually within the group of heritable or acquired skin diseases. Indeed, although elastic fiber fragmentation has been described in eye diseases such as conjunctivochalasis (with fiber fragmentation in the conjunctiva) or in primary open-angle glaucoma (fiber loss and fragmentation in the lamina cribrosa), these entities are clinically completely different from the ophthalmological features in PXE [50, 51]. Similarly, elastic fiber fragmentation has been described in certain tumor types, such as the papillary fibroelastoma, but these disorders lie beyond the scope of this paper [51]. 

### 5.1. Fibroelastolytic Papulosis

Fibroelastolytic papulosis (FEPN) encompasses a spectrum of two diseases reported as PXE-like papillary dermal elastolysis (PXE-PDE) and white fibrous papulosis of the neck (WFPN). Both can highly resemble the clinical skin lesions in PXE, characterized by yellowish papules which may coalesce into larger plaques. Contrary to PXE, these FEPN lesions may be pruritic and are not associated with other systemic symptoms [52]. 

#### 5.1.1. PXE-Like Papillary Dermal Elastolysis

**Figure 9:**
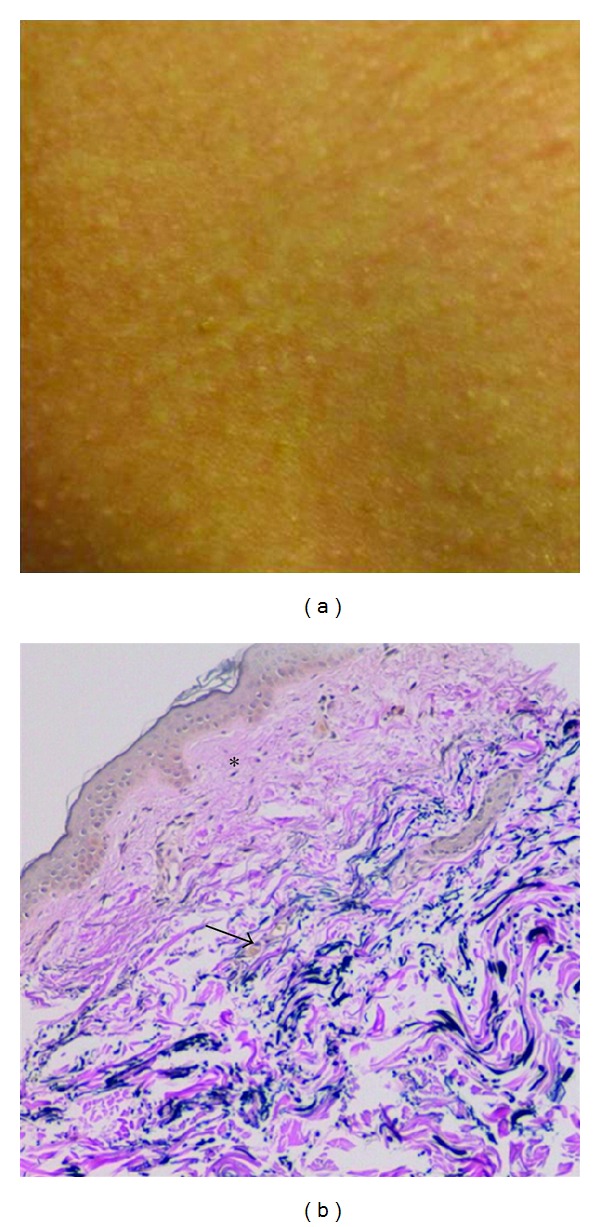
PXE-like papillary dermal elastolysis. Small flesh-colored papules can be seen in the neck region (a). LM examination reveals loss of elastic fibers in the papillary dermis and abnormal pattern in the reticular dermis (b, arrowed, ×20).

PXE-PDE is a very rare clinicopathological entity, which refers to an acquired disorder characterized by papules that resemble PXE clinically, and loss of elastic tissue in papillary dermis (Figure 9) [53, 54]. The disease typically affects women in late adulthood. With less than 40 cases reported, it has been suggested to be rather underdiagnosed [55]. Though its pathogenesis is not completely understood, the etiopathogenic factors considered include intrinsic skin aging, ultraviolet radiation, and abnormal elastogenesis [54, 55]. PXE-PDE may appear as multiple, asymptomatic or pruritic, yellow or skin-colored, nonfollicular, cobblestone-appearing papules that coalesce into large plaques distributed symmetrically over the supraclavicular, lateral, and posterior regions of the neck, the flexor aspect of the forearms, the axillae, the lower part of the abdomen, and the inframammary folds [54, 56]. 

Histopathologic examination of the affected skin shows an atrophic epidermis and band-like loss of elastic tissue in the papillary dermis (Figure 9(b)) [57]. Clumping and fragmentation of elastic fibers may also be seen [56]. In addition, melanophages can be observed in the papillary dermis, possibly as a consequence of subclinical junctional photodamage. Immunohistochemical staining may show loss of fibrillin 1 and 2, and microfibril-associated glycoprotein 1 and 2 in the papillary dermis [58]. 

#### 5.1.2. White Fibrous Papulosis of the Neck

WFPN is characterized by multiple, pale, discrete, nonfollicular lesions on the lateral and posterior neck with histologic evidence of fibrosis and variable loss of dermal elastic tissue. The lesions are asymptomatic and gradually increase in number. There is some clinical resemblance to PXE, but as in PXE-PDE there are no systemic complications such as angioid streaks in fundo [59–62]. The etiology is unknown, but in view of its late onset in life, the condition may be the result of age-related changes in dermal collagen [63]. 

Lightmicroscopy examination most prominently reveals dermal fibrosis consisting of areas of thickened collagen bundles in the papillary and midreticular dermis as well as loss of elastic tissue in the papillar and reticular dermis. The presence of fibrosis is the main feature to distinguish WFPN from PXE-PDE. Also complete absence of oxytalan fibers, an ECM component, has been noted [60, 62–64]. Electron microscopy may show a decrease in elastic tissue [65]. Remaining elastin fibers appear smaller, fragmented, and cribriform [62]. 

### 5.2. Late-Onset Focal Dermal Elastosis

Late-onset focal dermal elastosis (LOFDE) is a rare disorder of elastic tissue, characterized by a PXE-like yellowish papular eruption, with local accumulation of elastic fibers in the mid- and deep reticular dermis [66, 67]. The disease occurs mainly in elderly people and typically affects the sides of the neck and flexural areas, closely mimicking PXE from the clinical point of view [66]. The elderly onset of this disease suggests that LOFDE may be a disorder of aging. Occurrence of the lesions on the neck, antecubital and popliteal fossae is potentially significant; it has been hypothesized that elastic fiber turnover may be accelerated in these areas, which are naturally subjected to mechanical stress [67, 68]. Isolated fibroblasts from lesional skin show increased collagen and elastin messenger RNA suggesting that the disorder may be the result of increased elastin synthesis rather than a reduction in the degradation process [69]. 

The most prominent histopathologic feature is a local accumulation of normal-appearing elastic tissue or thick, interlacing elastic fibers in the mid- and deep reticular dermis without fragmentation, calcification, or phagocytosis of elastic fibers [69–71]. A hydroxyproline assay of lesional skin may show a modest increase in collagen content and a more substantial increase in elastin [72]. 

### 5.3. Perforating Calcific Elastosis

Perforating calcific elastosis (PCE), also referred to as “Localized acquired cutaneous pseudoxanthoma elasticum,” “Peri-umbilical perforating pseudoxanthoma elasticum (PPPXE),” or “Perforating periumbilical calcific elastosis,” is a noninherited, localized skin disease found most frequently in obese, multiparous, middle-aged women [73–76]. It is characterized by a yellowish, lax, well-circumscribed, reticulated or cobblestoned patch or plaque in the periumbilical region with keratotic papules scattered on its surface [74, 75, 77, 78]. The pathogenesis remains largely unknown, though it has been suggested to result from cutaneous trauma by obesity or ascites. 

**Figure 10:**
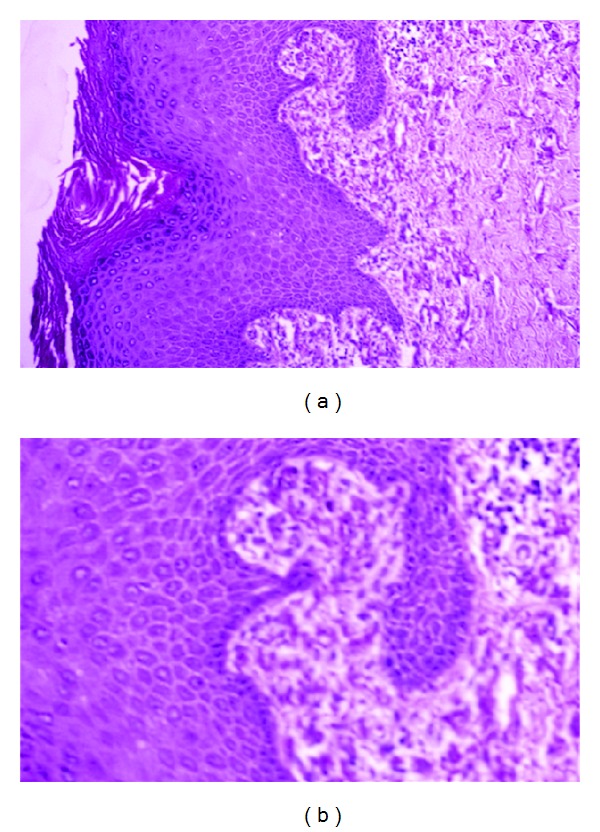
Perforating calcific elastosis. Clumping of short elastic fibers in the dermis can be observed (a, ×20; b, ×40).

As PXE, PCE is characterized histologically be degenerated elastic fibers that become infiltrated with calcium and lie predominantly in the middermis [79]. Hematoxylin-eosin-stained sections shows altered elastic fibers throughout the dermis that are short, thick, irregularly clumped, and basophilic (Figure 10). These calcified elastic fibers can be extruded to the skin surface through a channel lined by acanthotic epidermis, with multinucleated giant cells adjacent to the area of transepidermal elimination [80].

### 5.4. Buschke-Ollendorff Syndrome

The Buschke-Ollendorf syndrome (OMIM# 166700) is an autosomal dominant disease, characterized by disseminated connective tissue nevi and osteopoikilosis [81]. As these two main features may occur separately, it can be difficult to distinguish the skin lesions from fibroelastolytic elastosis, PXE, or papular elastorrhexis. The presenting symptom may be disseminated lesions on the trunk, upper arms, and thighs. The skeletal features, fascicular streaks, or condensations of the epiphysis and metaphysis of the long bones, are usually an incidental finding which often occur later than the skin features. In some patients, only one of the cardinal features is present [82]. The disease is caused by loss-of-function mutations in the *LEMD3* gene (OMIM# 607844), encoding an inner nuclear membrane protein [83].

**Figure 11:**
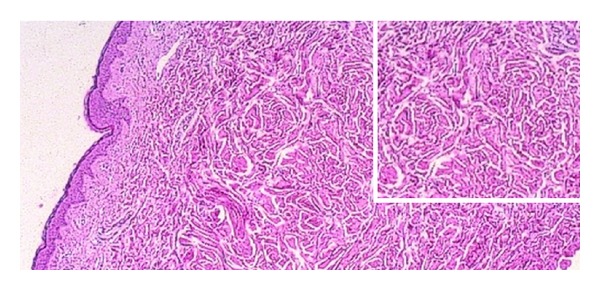
The Buschke-Ollendorf syndrome. LM image of dermatofibrosis lenticularis dissiminata (×20, insert ×40).

 Histologically, the lesions may either be elastin-rich (elastoma) or collagen-rich (dermatofibrosis lenticularis dissminata, Figure 11). For the elastomae, dermal elastic fibers may be increased in number and size, with hypertrophy on light microscopical exam. Electron microscopy can reveal altered electrolucent elastic fibers and decrease of microfibrillar components [84].

## 6. Related Dermatological Diseases

In this section, some differential diagnosis, which can occur in PXE patients or have a similar histological phenotype, will be discussed.

### 6.1. Elastosis Perforans Serpiginosa

Elastosis perforans serpiginosa (EPS; OMIM# 130100) is a rare skin condition of unknown aetiology characterized by hyperkeratotic papules and plaques, transepidermal elimination of abnormal elastic fibers, and focal dermal elastosis [67, 85]. Patient ages range from 5 to 89 years, but 90% of patients with EPS are younger than age 30, with approximately 75% of affected being male [86]. EPS has been postulated to be a focal irritation in the dermis, which may induce formation of epidermal and follicular channels to extrude the irritating agent. The disease can also occur in patients with an underlying connective tissue disorder, such as PXE or Ehlers-Danlossyndrome type IV (the vascular subtype) [87]. EPS can typically be induced by D-Penicillamine, used to treat Wilson disease. In the latter, patients develop skin lesions that may be highly resemblant of PXE, which is sometimes referred to as pseudo-PXE [88]. In these cases, discontinuation of the drug usually leads to improvement of the lesions. 

**Figure 12:**
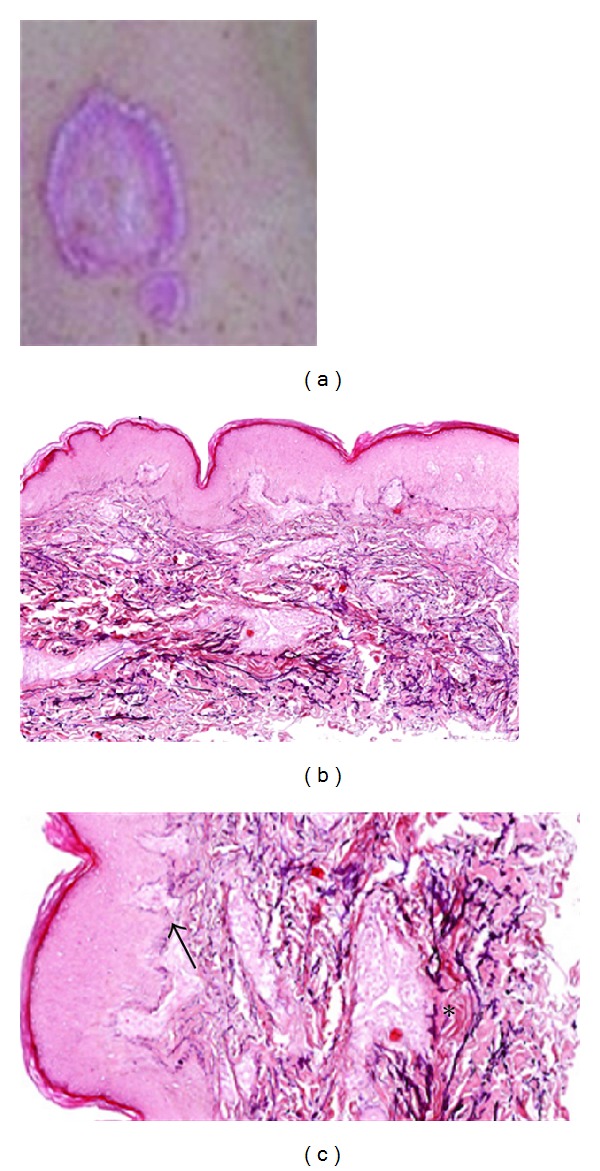
Elastosis perforans serpiginosa. Hyperkeratotic plaques of papules in the neck of a patient (a). LM examination with degenerated elastic fibers (b, c, asterisk) and transepidermal perforating canals (b, c, arrowed, ×20 and ×30).

Symptoms of EPS can usually appear in the neck and on the face and, less frequently, the upper and lower extremities or trunk with pruritic, erythematous or skin-colored hyperkeratotic papules and plaques with central scaling, hypopigmentation, and atrophy. Papules are typically distributed in a serpiginous pattern (Figure 12(a)). 

The most prominent histopathological feature of EPS is the presence of narrow transepidermal or perifollicular perforating canals that extend upward in a straight or corkscrew pattern from the dermis and contain a mixture of degenerated eosinophilic elastic fibers, basophilic debris, and inflammatory cells (Figures 12(b) and 12(c)). Significantly increased amount and thickness of papillary dermal elastic tissue is also noticed [86]. A chronic inflammatory infiltrate with granuloma-forming multinucleated giant cells may be present [89]. The epidermis may be acanthotic and hyperkeratotic. LM and EM of Penicillamine-induced EPS reveal a characteristic “lumpy-bumpy” or “bramble-bush” appearance of elastic fibers and elastic tissue changes are observed in both lesional and nonlesional skin, which distinguishes it from other types of EPS [90–94]. Transepidermal elimination may also be seen in some forms of acquired PXE; however, the mineralization of elastic fibers characteristic of PXE is absent in EPS. 

### 6.2. Papular Elastorrhexis

Papular elastorrhexis (PE) is a sporadic dermatosis, mostly occurring in adolescence, characterized by asymptomatic firm, nonfollicular, 1–5 mm diameter, well-demarcated white papules distributed evenly over the trunk including the chest, abdomen, back, shoulders, and upper extremities [85, 95–98]. Though the cause and pathogenesis of PE is mostly unknown, recent studies suggest that PE can occur in both acquired and familial forms [99]. It is regarded as a variant of elastic tissue nevi though controversy exists [98]. 

PE causes substantial fragmentation or nearly complete loss of elastic tissue in the reticular dermis [99–102]. A perivascular lymphohistiocytic infiltrate in the superficial and deep dermis has been reported. Collagen bundles can be thickened and homogenized, or normal [99]. 

### 6.3. Upper and Middermal Elastolysis

Upper and middermal elastolysis are rare disorders, which are both characterized by a significant loss of dermal elastic fibers. Though they may be highly resemblant clinically, the main differences can be noted histologically in the location of the absent elastic fibers but also in severity of ultrastructural damages [103].

#### 6.3.1. Upper Dermal Elastolysis

Upper dermal elastolysis is characterized by an eruption of many small papules on the neck, shoulder, upper chest, and upper back region and selective loss of elastic tissue in the papillary dermis [103, 104]. It has been suggested that lysis of elastic tissue may be a primary process in activation of dermal phagocytes that recognize fragments of elastic fibers as foreign. Alternatively, activation of dermal phagocytes may be a primary inflammatory process that leads to elastolysis and engulfment of both normal-appearing and degraded elastic fibers [103]. 

Histopathology is characterized by complete loss of elastic fibers in the upper dermis including the papillary dermis, whereas middermal fibers are intact. Electron microscopy reveals a loose assembly of elastic fibrils and electron-dense substance aggregated between the loosely bound subunits. Elastophagocytosis of normal-appearing and abnormal fibers is also observed [103]. 

#### 6.3.2. Middermal Elastolysis

Middermal elastolysis typically occurs in healthy young or middle-aged women and is characterized by focal loss of elastic tissue in the midreticular dermis. The disease manifests clinically as multiple, discrete, wrinkled, flesh-colored, soft papules, some with a central umbilication, were present on the abdomen and lower back [104, 105]. Many cases appear to be induced or worsened by ultraviolet light exposure [106]. 

**Figure 13:**
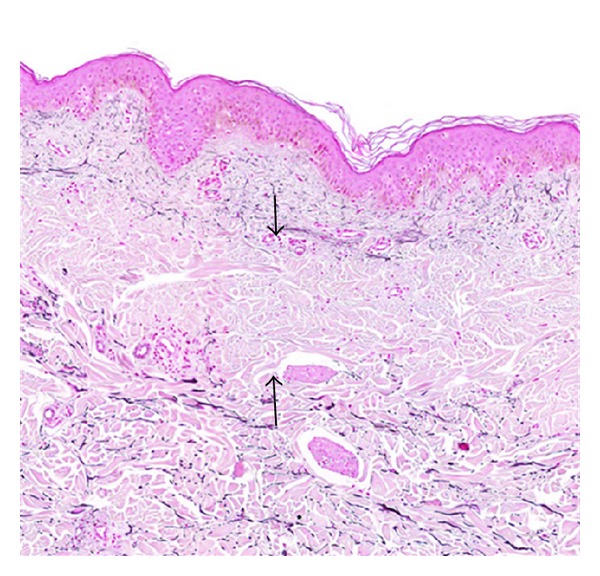
Middermal elastolysis. Band-like loss of elastic fibers in the midreticular dermis, sparing the papillary and deep reticular dermis (×20).

**Figure 14:**
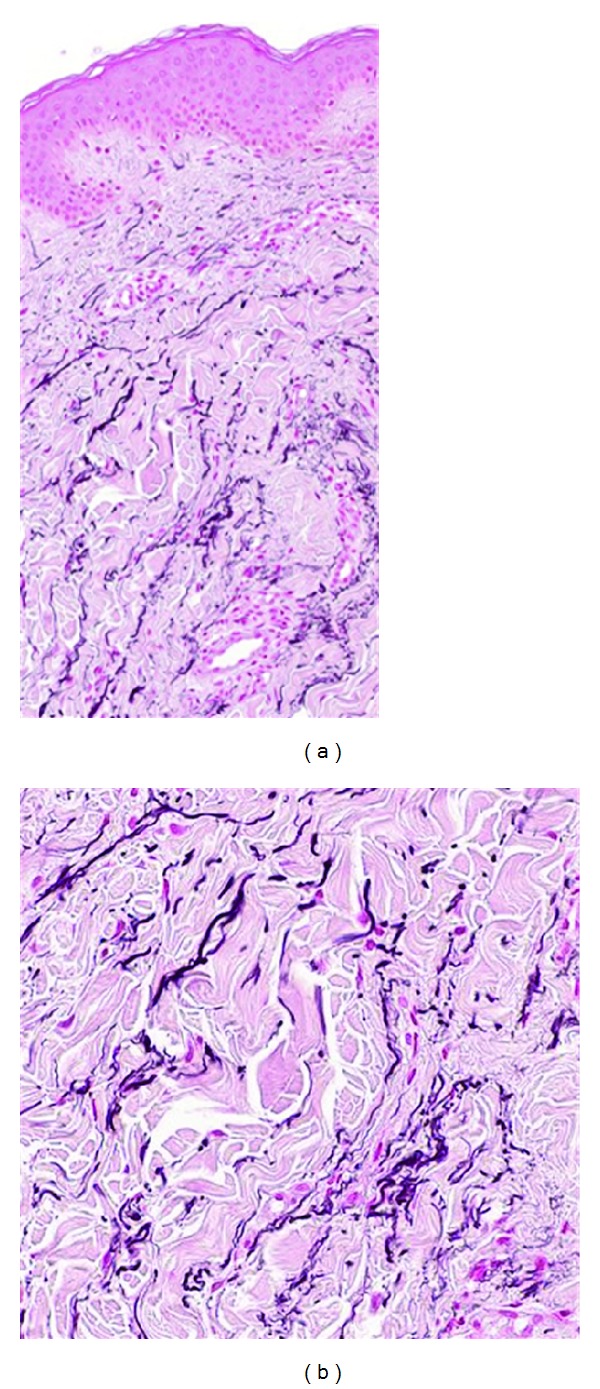
Linear focal elastosis. Accumulation of fragmented elastotic material within the papillary dermis (a, b) and transcutaneous elimination of elastotic fibers (×20 and ×40).

On histological examination, a selective absence of elastic fibers (Figure 14) in the middermis can be observed (Figure 13). Additionally, perivascular lymphocytic, monocytic, or neutrophilic infiltrates and phagocytosis of elastin by multinucleated giant cells can be seen [107–112]. Electron microscopy studies have reported phagocytosis of degenerated elastic fibers by macrophages [107, 111, 113–115], as well as loose assembly of skeleton fibrils and irregular aggregations of dense substance [107, 110, 115–117]. Immunohistochemistry shows absent immunoreactivity of elastin. Stainings for MMP-9 can be positive in epidermal keratinocytes and in large multinucleated cells in the affected dermis [118]. 

### 6.4. Linear Focal Elastosis

Linear focal elastosis (LFE) is a disorder of elastic fibers characterized by palpable, hypertrophic, yellowish, linear striae distributed horizontally over the mid and lower back, thighs, arms and breasts, and an increase in abnormal elastic tissue [119–121]. Reports on occurrence show that men are more prone than women. Although the pathogenesis of LFE remains unknown, its association with striae distensae may suggest involvement of a process analogous though not identical to keloidal repair [120].

Lightmicroscopical examination can reveal massive, well-demarcated basophilic fibers and increased elastic tissue staining; elastic fibers in the subpapillary to lower reticular dermis appear fragmented and clumped [122–125]. Electron microscopy may show fragmentation of elastic tissue, as well as the presence of microfibrillar, granular components, and elastin, aggregated in various stages of maturation [122–124]. Elastin, fibrillin-1, fibrillin-2, and microfibril-associated glycoprotein-1 and 4 may be decreased in or absent from the papillary dermis of lesional skin [126]. Recently, immunohistochemistry in LFE showed absence of TGF*β* staining, suggesting that the pathogenesis of LFE is still different from TGF*β*-dependent keloid development [120]. 

### 6.5. Elastoderma

Elastoderma is an exceedingly rare condition presenting with an acquired localized laxity of skin resembling cutis laxa, with an abundance of pleomorphic elastic tissue in the dermis [127, 128]. It has been suggested that the abundance of elastic tissue results from increased synthesis, as evidenced by the presence of active fibroblasts with prominent rough endoplasmic reticulum [127–129]. It has been speculated that elastoderma may be a localized disorder of elastin synthesis analogous to localized scleroderma (Morphea) resulting from excessive collagen distribution [67]. 

Histologic examination of specimens from the affected area can reveal increased masses of intertwined thin, elastic fibers without calcification in the papillary and upper reticular dermis [127]. Electron microscopic examination demonstrates irregular deposition of elastic material at the periphery of elastic tissue fibers, with grape-like globular structures [127]. 

### 6.6. Calcinosis Cutis

Calcinosis cutis refers to a group of disorders featuring pathologic calcification, generally calcium phosphate deposits, of the skin and soft tissues [130]. Calcinosis cutis can occur in a variety of systemic and localized conditions and is classified into 4 major types according to etiology: dystrophic, metastatic, iatrogenic, and idiopathic; among these, dystrophic calcinosis cutis is most common. A few rare types have been variably classified as dystrophic or idiopathic [119]. These include calcinosis cutis circumscripta, calcinosis cutis universalis, tumoral calcinosis, and transplant-associated calcinosis cutis. Calcinosis cutis circumscripta tends to arise in second half of life, calcinosis cutis universalis occurs in the second decade of life, and the tumoral calcinosis usually arises in the first or second decade of life. The pathogenesis of calcinosis cutis is not completely understood, although metabolic and physical factors are pivotal in the development of most cases of calcinosis. 

**Figure 15:**
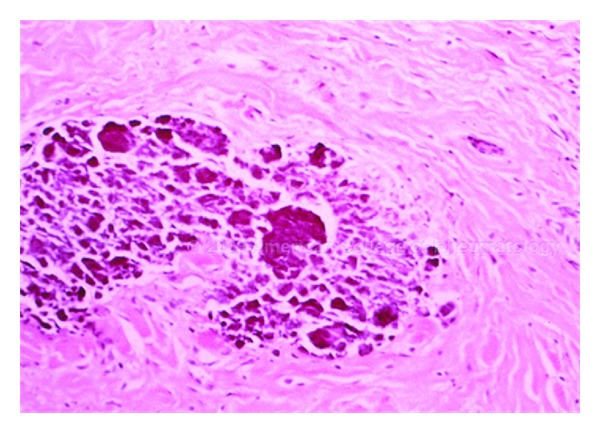
Dystrophic calcinosis cutis with large, basophilic-stained calcium deposits in the dermis (×40).

On skin biopsy, granules and deposits of calcium are seen in the dermis, with or without a surrounding foreign-body giant cell reaction (Figure 15). Alternatively, massive calcium deposits may be located in the subcutaneous tissue. In areas of necrosis, calcium deposition is frequently found within the walls of small- and medium-sized blood vessels [130]. 

## 7. Conclusion and Summary

The histological examination of the skin is currently still the golden standard to make a diagnosis of PXE. Because of the high mutation uptake of molecular analysis of the *ABCC6* gene, a skin biopsy is currently not always performed. However, in the absence of either two causal *ABCC6* mutations or of unequivocal systemic features compatible with PXE, a histological evaluation of the skin lesions still remains the most important diagnostic step towards a definite diagnosis. Indeed, the histological features—either on LM, EM or both—are capable of distinguishing most of the disorders in the differential diagnosis of cutaneous PXE, even when clinically highly resemblant or identical.  Table 1 summarizes the most important differential diagnoses of PXE and their histopathological clues. In those patients—for example, suffering from a haemoglobinopathy—where clinical and histological findings are identical to *ABCC6*-related PXE, additional blood examinations can be performed to confirm the diagnosis. 

## Figures and Tables

**Table 1:** Histopathological clues of PXE and related disorders.

Disease	Histopathological clue(s)
Pseudoxanthoma elasticum	LM: Mid-dermal calcification and fragmentation of elastic fibers
	EM: Mineralization in elastic fiber core
PXE-like disease with coagulation deficiency	LM: Middermal calcification and fragmentation of elastic fibers
	EM: Mineralization in elastic fiber periphery
Haemoglobinopathies	LM: Middermal calcification and fragmentation of elastic fibers
	EM: Mineralization in elastic fiber core
PXE-like papillary dermal elastolysis	LM: Selective elastic tissue elimination in the papillary dermis and presence of melanophages
White fibrous papulosis of the neck	LM: Dermal fibrosis in papillary and mid-reticular dermis
	EM: Decrease of elastic fibers; fragmentation of remaining fibers
Late-onset focal dermal elastosis	LM: Accumulation of elastic fibers in mid- and reticular dermis without fragmentation or calcification
Perforating calcific elastosis	LM: Middermal calcification and degeneration of elastic fibers with transepidermal elimination
Buschke-Ollendorff syndrome	LM: Increased amount of hypertrophic elastic fibers in dermis
	EM: Altered translucent elastic fibers
Elastosis perforans serpiginosa	LM: Transepidermal or perifollicular perforating canals
Papular elastorrhexis	LM: Thickening of collagen bundles next to loss and fragmentation of elastic fibers
Upper dermal elastolysis	LM: Complete loss of elastic fibers in the upper dermis
Middermal elastolysis	LM: Complete absence of elastic fibers in the middermis
Linear focal elastosis	LM: Massive basophilic fibers; clumping of elastic fibers in papillary dermis
Elastoderma	LM: Increased, intertwining thin elastic fibers in papillary and upper reticular dermis
Calcinosis cutis	LM: Deposits of calcium in the dermis
